# Supplementary material for: Transcription Factor NFE2L1 Decreases in Glomerulonephropathies after Podocyte Damage
Source: Cells. 2023 Aug 29;12(17):2165. doi: 10.3390/cells12172165 (PMC10487238; doi:10.3390/cells12172165)
Supplement: Supplementary file 1 [file cells-12-02165-s001.zip › cells-2433823-supplementary.pdf]

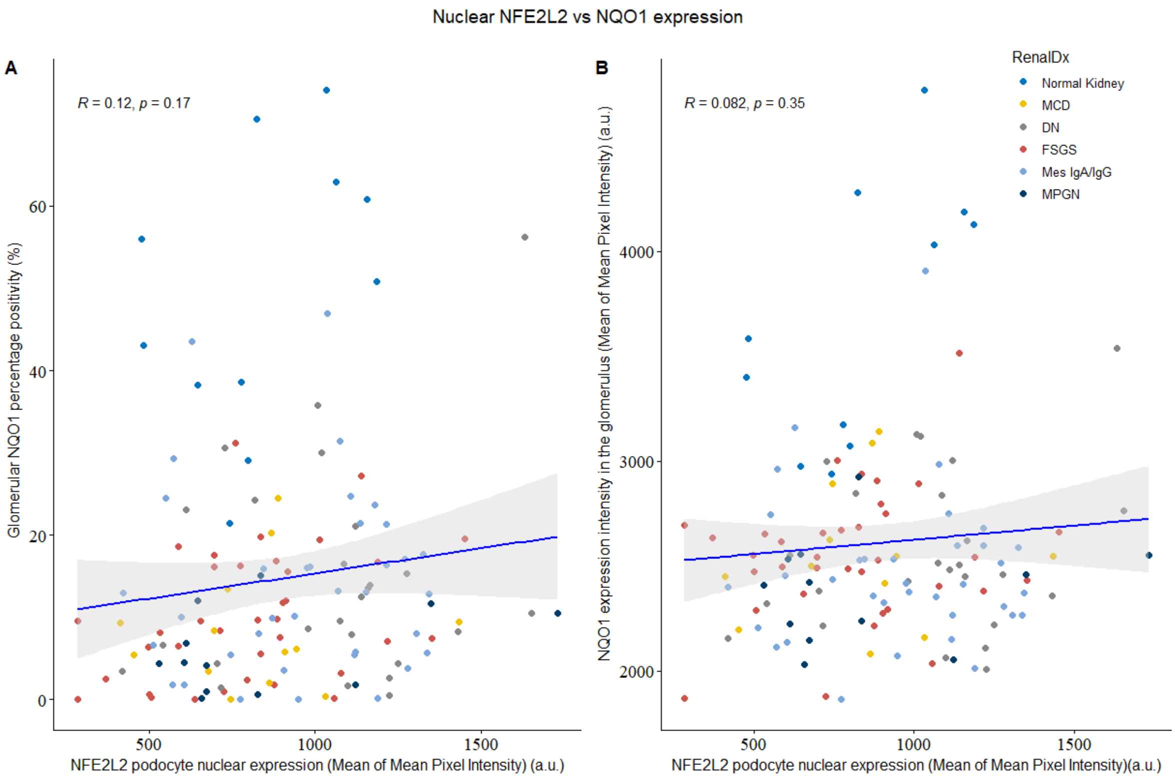

Figure S1. NFE2L2 podocyte nuclear expression vs glomerular NQO1 A) Comparison between NFE2L2 podocyte nuclear expression and Glomerular NQO1 percentage positivity shows no significant correlation (R=0.12, P<0.17). B) Comparison between NFE2L2 podocyte nuclear expression and NQO1 expression intensity shows significant correlation (R=0.66, P<2.2e-16). Correlation was evaluated by Spearman's correlation coefficient.

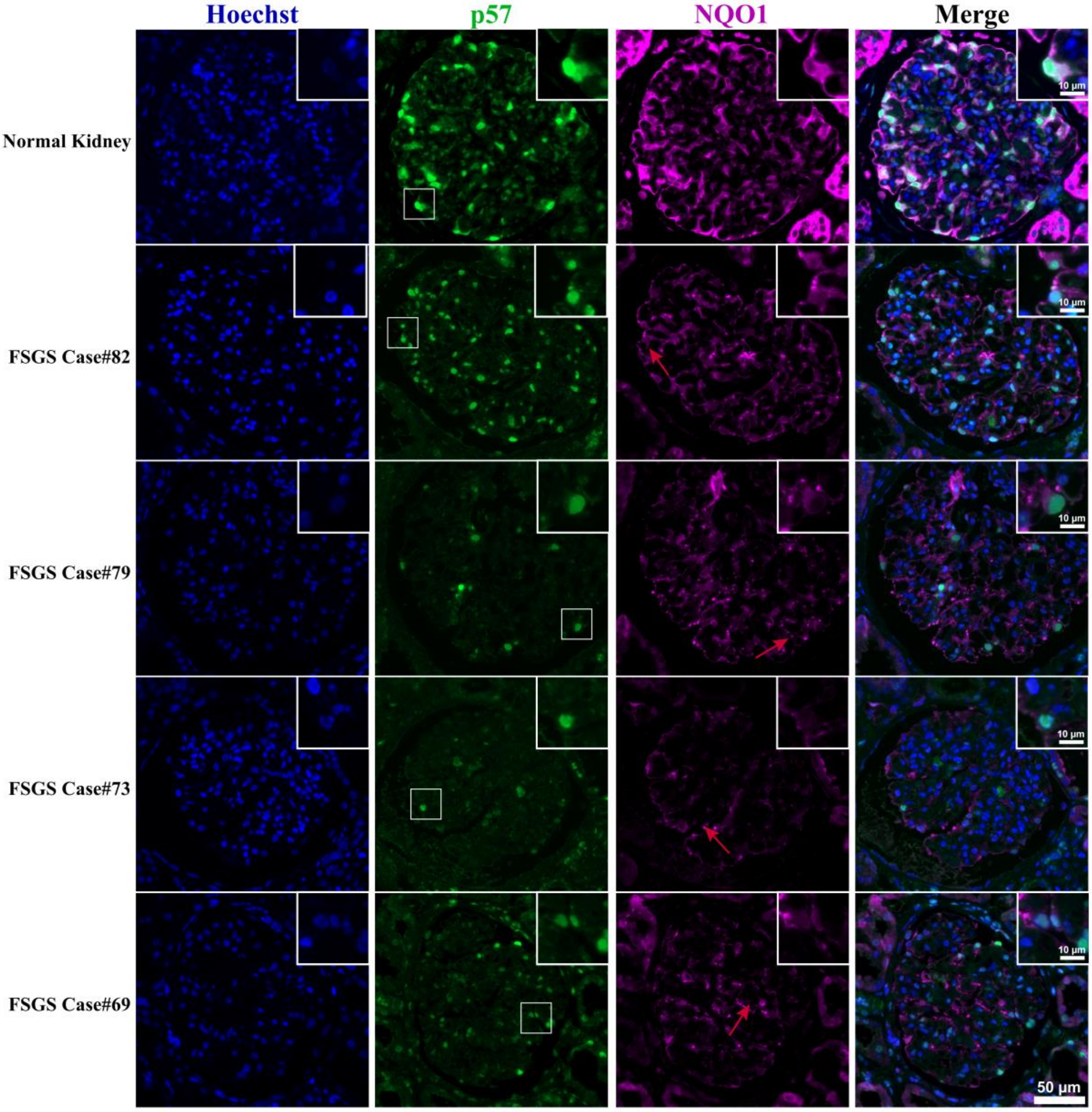

Figure S2. p57 and NQO1 expression in normal and kidney disease biopsies. A representative image of glomeruli from normal and kidney disease biopsies. The sections were multiplexed with Hoechst (blue), p57 (green), and NQO1 (purple) antibodies. The inset image at the top right corner is a zoomed image of the white boxed region of interest representing a podocyte Magnification 20x with scale bar representing 50 µm and 10 µm.

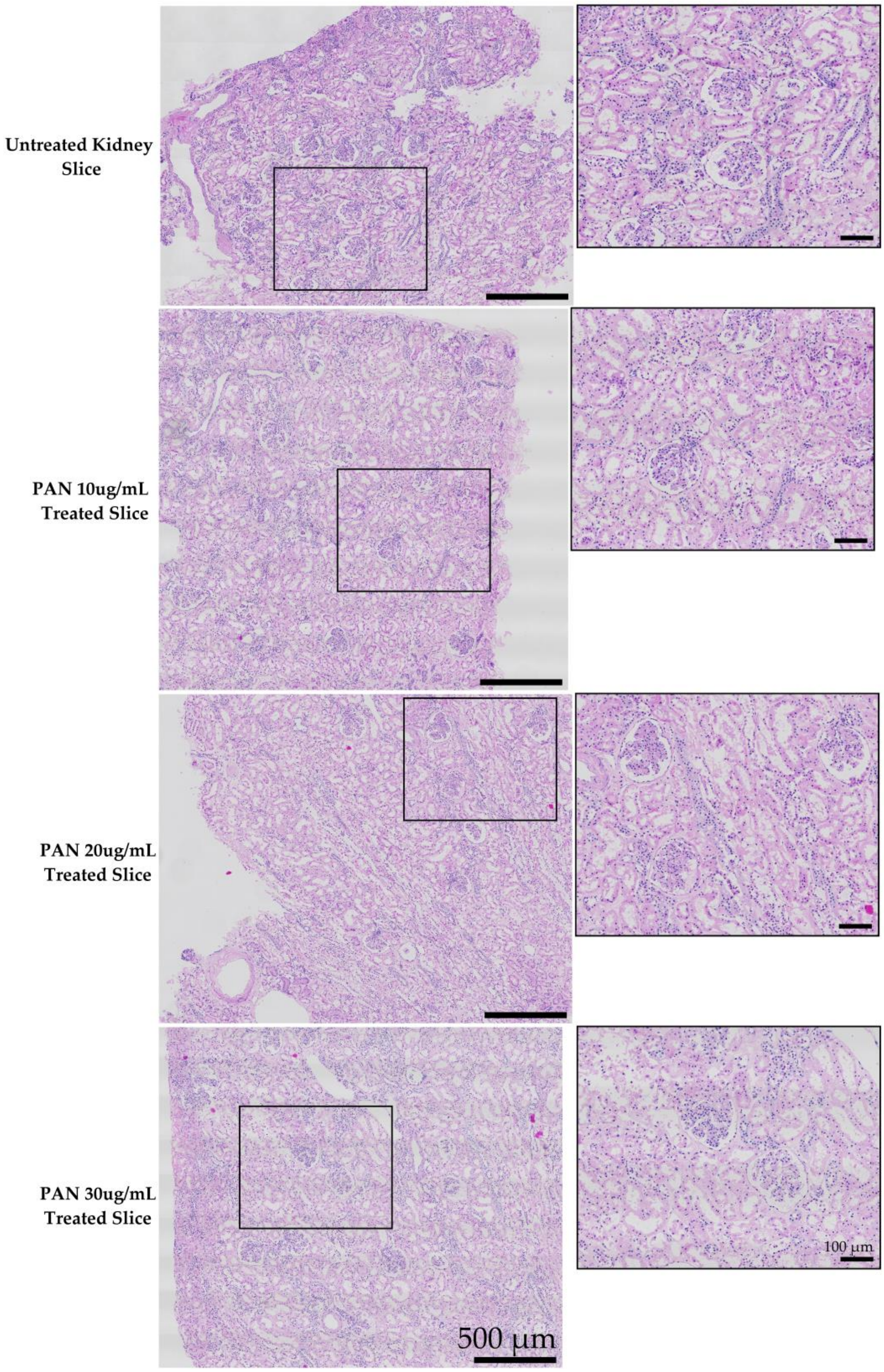

Figure S3. H&E of ex-vivo kidney slices. A) x5 magnification of H&E kidney slices, showing intact glomeruli, bar represents 500 μm. B) H&E image of the glomerulus from ex-vivo kidney slice with attached podocytes and intact glomerulus. Image x20 magnification, scale bar represents 100 μm.

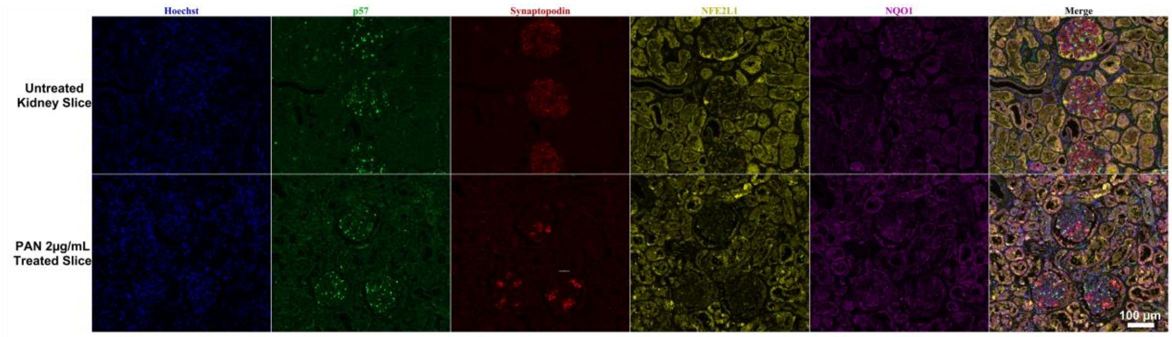

Figure S4. NFE2L1, NQO1 and Synaptopodin expression in untreated and PAN treated *ex-vivo* human kidney slices. A representative image of glomeruli from ex-vivo kidney slices, untreated slices from normal kidney parenchyma and 20 μg/mL treated for 24 h. The sections were multiplexed with Hoechst (blue), p57 (green), synaptopodin (red) NFE2L1 (yellow) and NQO1 (purple) antibodies. Magnification 20x with scale bar representing 100 μm.

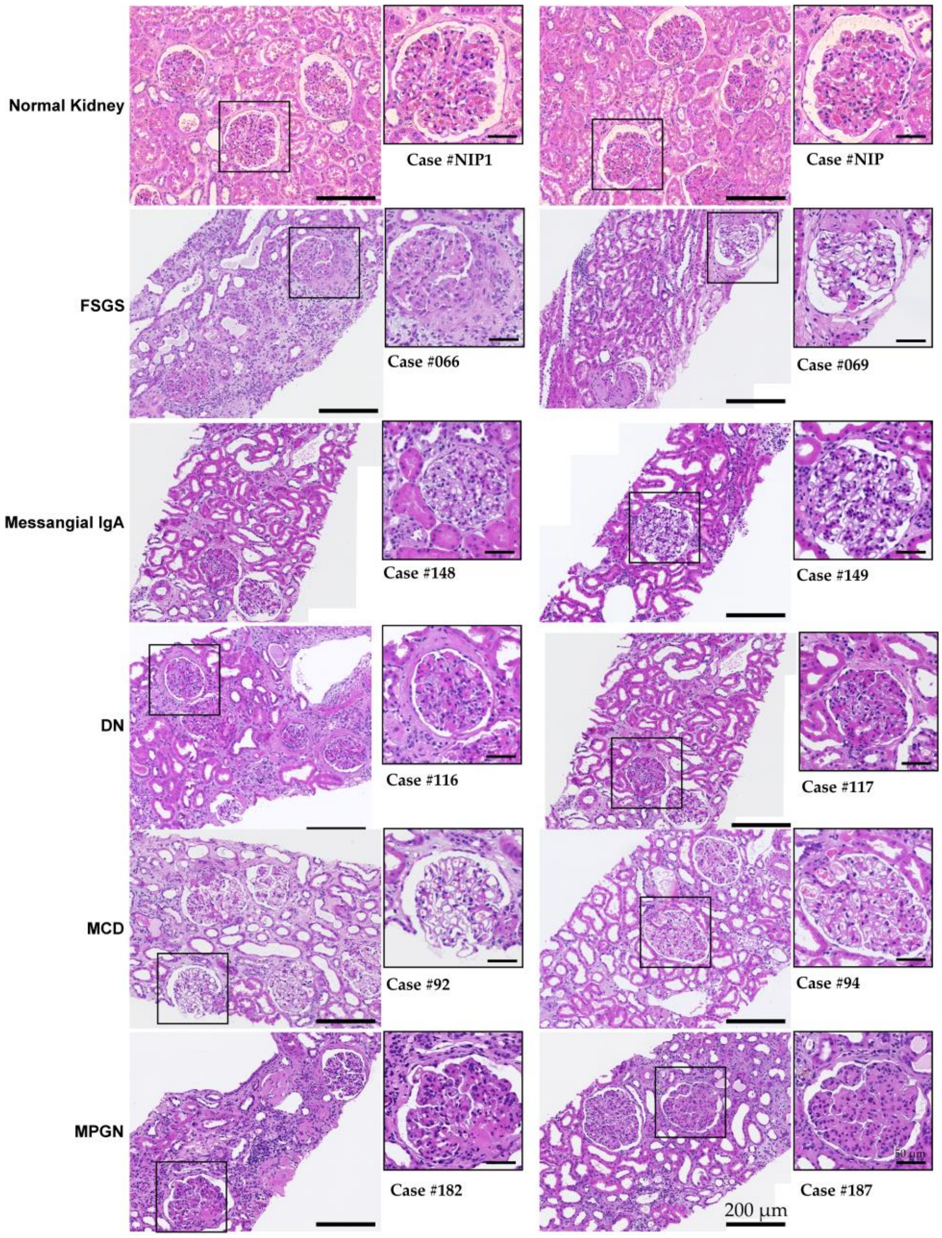

Figure S5. Representative H&E images from normal cases and kidney disease biopsies, The 20x magnification image showed partial biopsies, scale bar represents 200 µm. The zoomed images show a representation of glomeruli analysed in the image analysis, scale bar represents 50 µm.

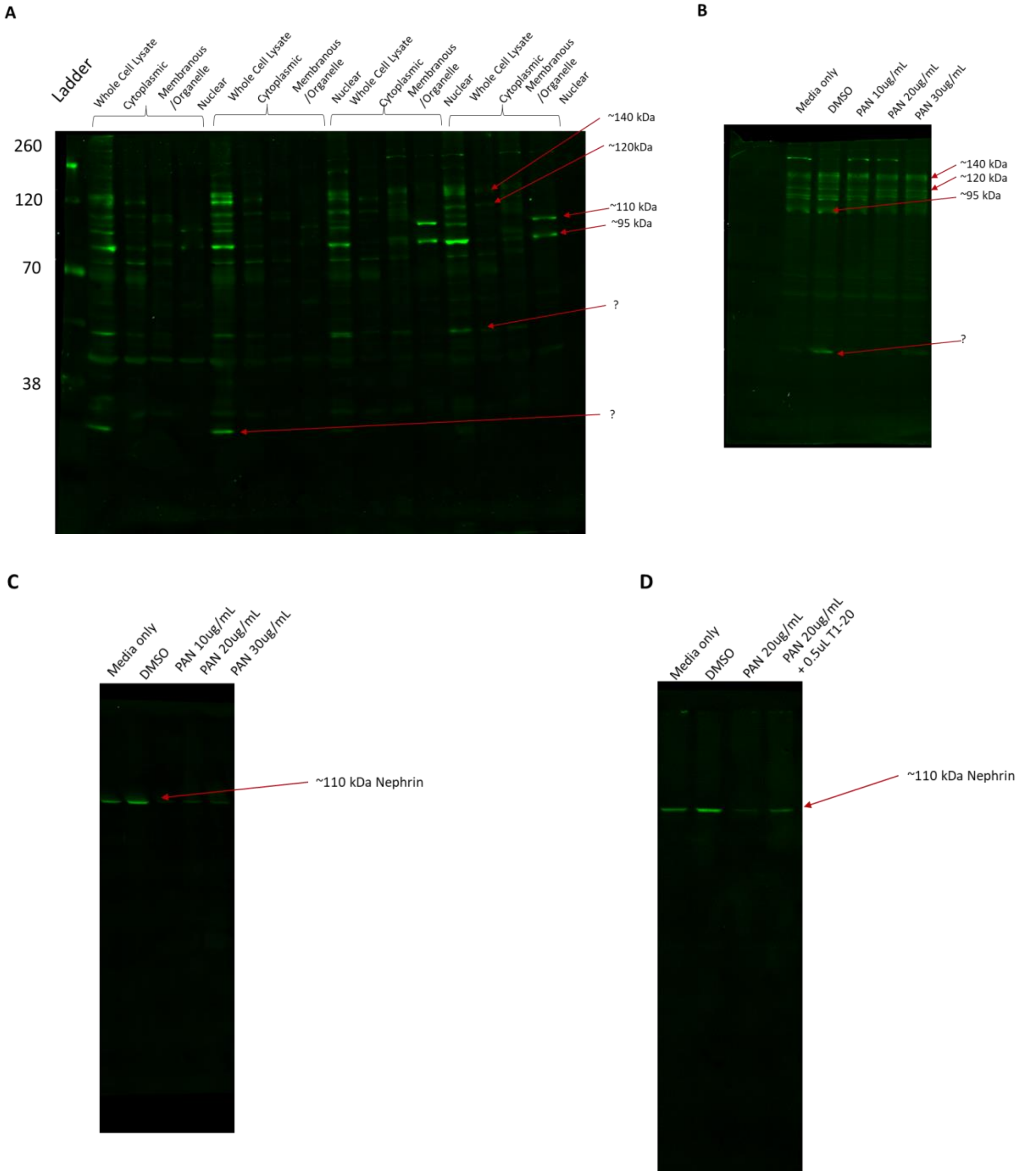

Figure S6 . Whole western blot images A) Podocyte cell fractionation and immunoblotting of NFE2L1: The whole western blot images show the expression of NFE2L1 in podocytes after cell fractionation. NFE2L1 staining of PAN (protamine sulfate)-treated podocytes is also presented. B) NFE2L1 protein expression: The western blot displays NFE2L1 protein expression in 14-day differentiated podocyte samples. The samples include untreated control, DMSO, and those treated with different concentrations (10 µg/mL, 20 µg/mL, and 30 µg/mL) of PAN for 24 hours, ± T1-20 (a reference compound). C) Nephrin protein expression: The western blot illustrates nephrin protein expression in 14-day differentiated podocytes treated with varying concentrations of PAN (10 µg/mL, 20 µg/mL, and 30 µg/mL) for 24 hours.D) Representative image of nephrin protein expression: A representative image depicting nephrin protein expression in 14-day differentiated podocyte samples. The samples consist of untreated control, DMSO, and those treated with 20 µg/mL PAN for 24 hours, ± T1-20.

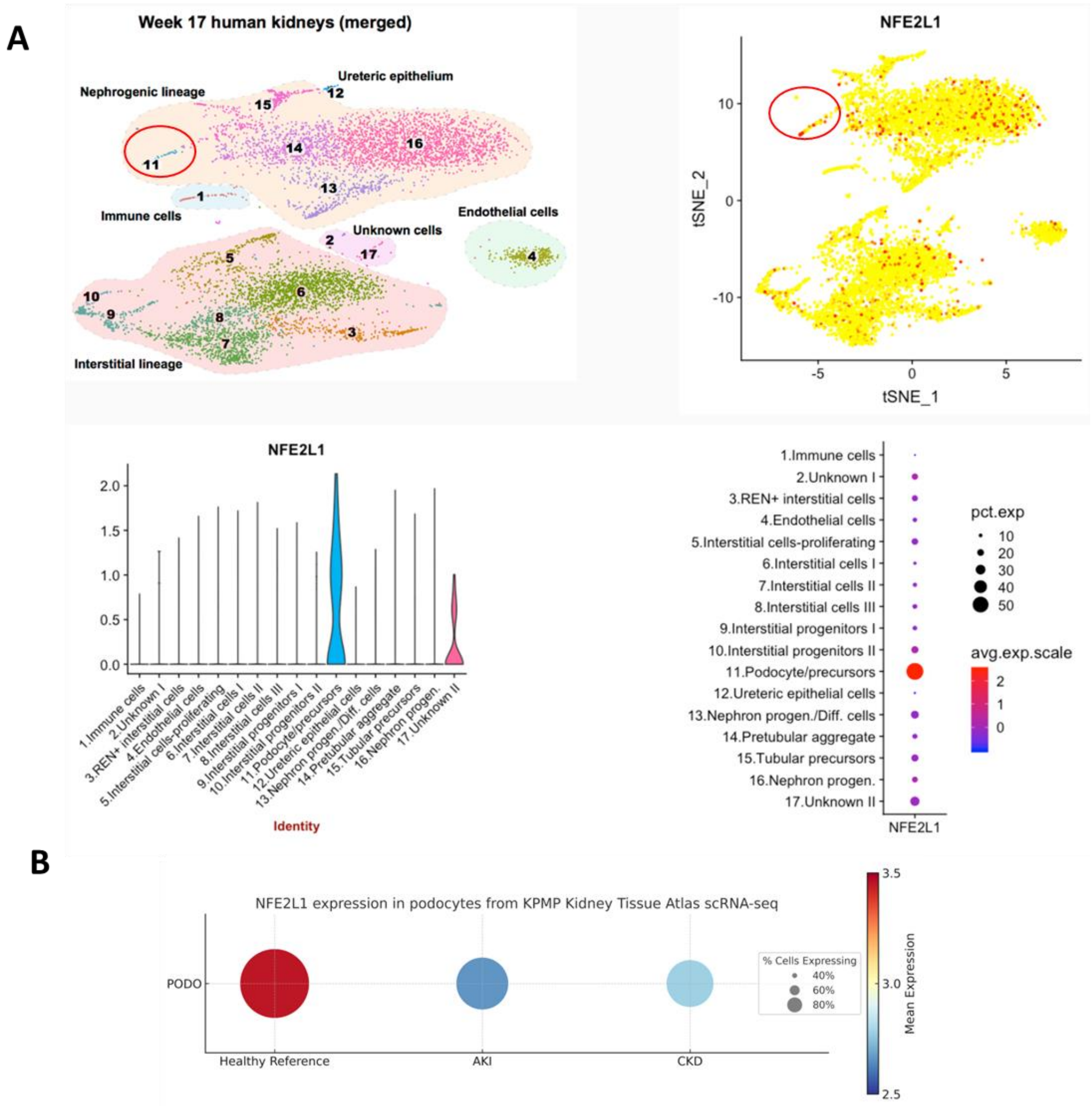

Figure S7. A) Analysis of nephron lineage cells using single-cell RNA-seq, visualized in a t-SNE plot. The heatmap illustrates NFE2L1 expression, highlighting an increase in expression within podocyte precursors. Expression maps further reveal that NFE2L1 gene expression is primarily present in both podocytes and their precursors, as evidenced by the single-cell RNA study of the human nephrogenic niche by Lindström et al., 2018. B) Analysis of scRNA-seq data from the KPMP consortium, showing RNA expression in podocytes from single-cell analyses of Healthy, Acute Kidney Injury (AKI), and CKD states. Data accessed from the KPMP Atlas (https://atlas.kpmp.org/explorer/) on 06/08/2023.

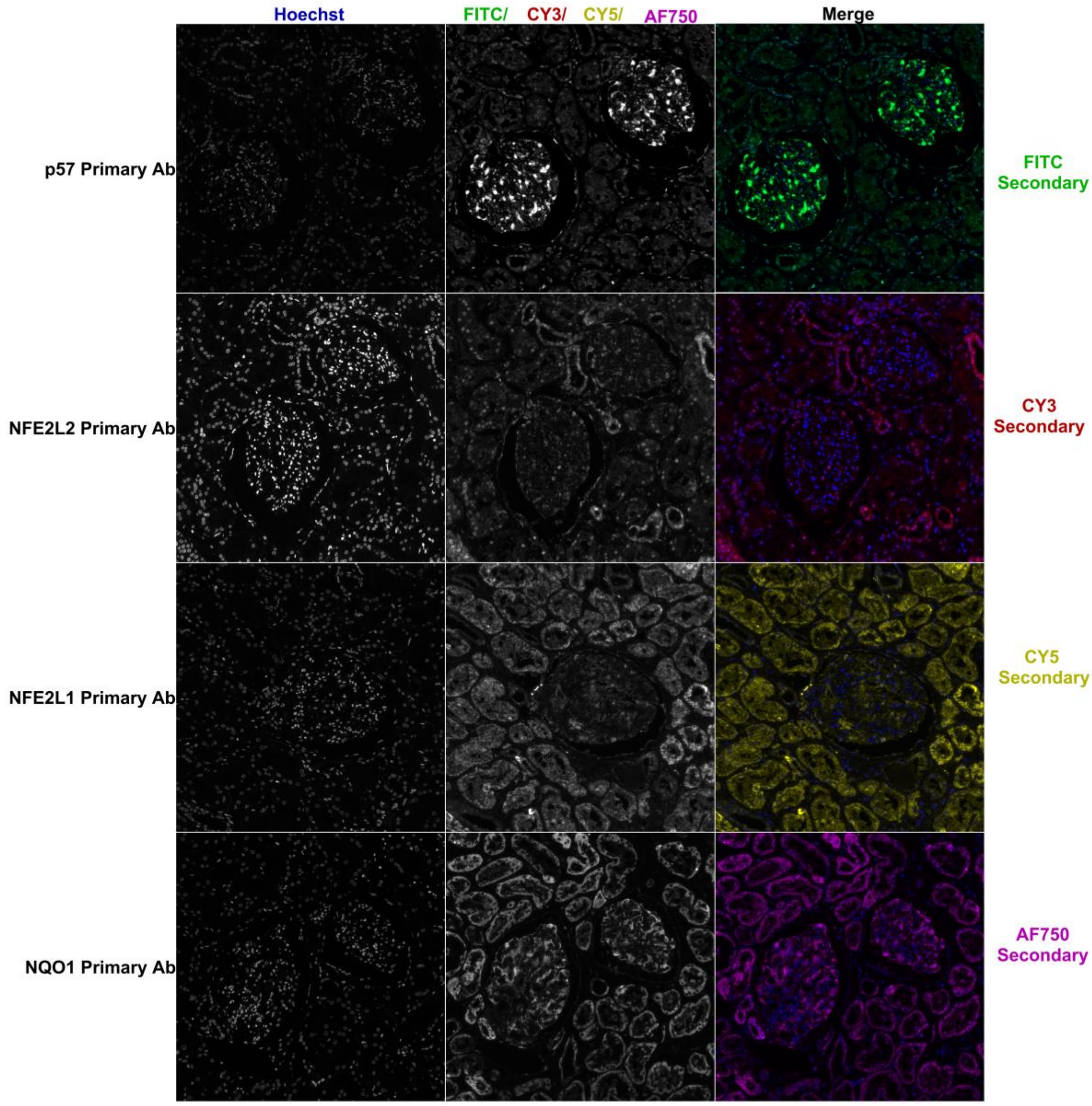

Figure S8. Representative images showing the optimization of Hoechst and single primary antibody immunofluorescence staining. This optimization process was conducted prior to proceeding with multiplexing to validate the immunospecificity of the antibodies used. The primary antibodies used were p57 (visualized with FITC secondary antibody), NFE2L2 (visualized with Cy3), NFE2L1 (visualized with Cy5), and NQO1 (visualized with AF750).
